# Pneumopericardium: a rare case of cardiorespiratory arrest

**DOI:** 10.31744/einstein_journal/2019AI4516

**Published:** 2019-04-29

**Authors:** Cristian Tedesco Tonial, Pedro Celiny Ramos Garcia, Julia Victora, Caroline Abud Drumond Costa, Joanne Sausen Velasques, Marcio Abelha Martins

**Affiliations:** 1 Pontifícia Universidade Católica do Rio Grande do Sul, Porto Alegre, RS, Brazil.; 2 Universidade de Santa Cruz do Sul, Santa Cruz do Sul, RS, Brazil.

A 37-days-old baby boy, previously healthy and without intercurrences during perinatal period, who was admitted in pediatric intensive care unit with acute ventilator insufficiency because of viral bronchiolitis due to respiratory syncytial virus. The patient underwent a tracheal intubation by clinical deterioration, a rapid sequence intubation using fentanyl, ketamine and succinylcholine. During the procedure, he had cough, chest rigidity, reduction of respiratory sounds and poor tissue perfusion. His clinical picture worsened and resulted in cardiorespiratory arrest in asystole that was reverted with chest compressions and two doses of intravenous epinephrine. Subsequently, the patient showed a new cardiorespiratory arrest, and he was under risk for hypertensive pneumothorax. A relief thoracentesis was carried out and significant improvement was observed in respiratory sound and signs of poor peripheral perfusion. After bilateral thoracic drainage the patient had ventilator parameters reduced, however, this reduction remained with impaired tissue perfusion, mottled skin, fine pulses, hypotension, and cardiac auscultation with hypophonesis sound. The chest radiology exam confirmed hypertensive pneumopericardium (Figure 1) that resolved with pericardiocenthesis and drainage of 40mL of pericardium air space (Figure 2). The patient had good clinical progress after underwent the procedure, remained in mechanical ventilation for 6 days, and he was discharged 13 days after the procedure without apparent sequelae.

**Figure 1:**
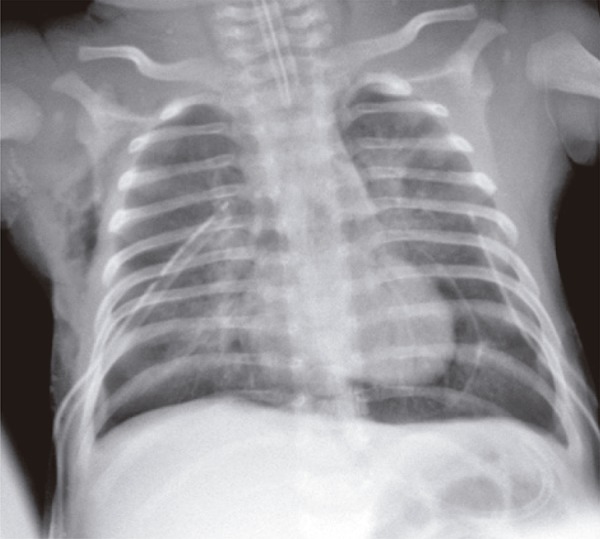
Pneumopericardium

**Figure 2:**
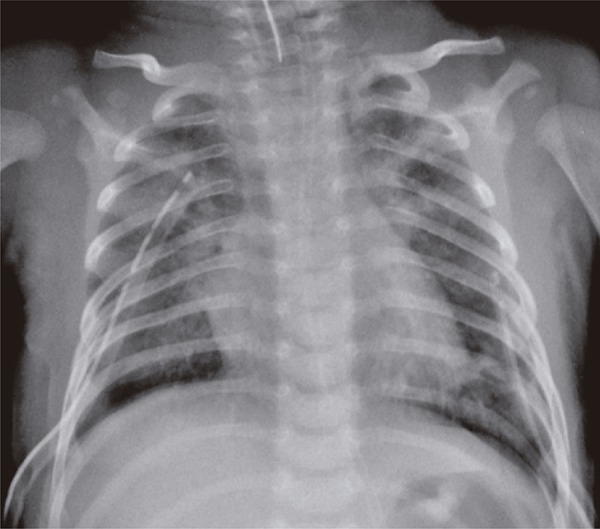
Pneumopericardium drainage after pericardiocentesis

Hypertensive pneumopericardium is a rare condition that can be related with positive-pressure ventilation, laryngeal obstruction, acute or severe asthma or closed chest trauma.^(^ ^1^ ^)^ Mortality rates can reach 80% in neonates.^(^ ^2^ ^,^ ^3^ ^)^ This condition has a clinical feature of cardiac tamponade and a differential diagnosis is pneumothorax.^(^ ^3^ ^)^ Premature patients with hyaline membrane disease who underwent mechanical ventilation are at higher risk for this disease, however, reports exist on spontaneous pneumopericardium in healthy infants, and in those who develop bronchiolitis by respiratory syncytial virus.^(^ ^4^ ^,^ ^5^ ^)^ In this case, although epinefrin administration have promoted temporary circulation support, the rapid diagnosis and resolution of the cardiac tamponade via pericardiocentesis contributed to the good evolution of the patient.^(^ ^6^ ^)^

## References

[1] el Gamel A, Barrett P, Kopff G (1994). Pneumopericardium: a rare cause of cardiac tamponade in an infant on a positive pressure ventilation. Acta Paediatr.

[2] Kyle A, Veldtman G, Stanton M, Weeden D, Baral V (2012). Barotrauma-associated posterior tension pneumomediastinum, a rare cause of cardiac tamponade in a ventilated neonate: a case report and review of the literature. Acta Paediatr.

[3] Neal MD, Hackam DJ (2011). Tension pneumopericardium in an infant. Surgery.

[4] Suresh P, Tagare A, Kadam S, Vaidya U, Pandit A (2011). Spontaneous pneumopericardium in a healthy full-term neonate. Indian J Pediatr.

[5] Fantacci C, Ferrara P, Franceschi F, Chiaretti A (2017). Pneumopericardium, pneumomediastinum, and pneumorrachis complicating acute respiratory syncytial virus bronchiolitis in children. Eur Rev Med Pharmacol Sci.

[6] Levin AI, Visser F, Mattheyse F, Coetzee A (2008). Tension pneumopericardium during positive-pressure ventilation leading to cardiac arrest. J Cardiothorac Vasc Anesth.

